# Association of Glycemic Indices (Hyperglycemia, Glucose Variability, and Hypoglycemia) with Oxidative Stress and Diabetic Complications

**DOI:** 10.1155/2020/7489795

**Published:** 2020-10-12

**Authors:** Eleftheria Papachristoforou, Vaia Lambadiari, Eirini Maratou, Konstantinos Makrilakis

**Affiliations:** ^1^First Department of Propaedeutic Internal Medicine, National and Kapodistrian University of Athens Medical School, Laiko General Hospital, Athens, Greece; ^2^Second Department of Internal Medicine, Research Unit and Diabetes Centre, National and Kapodistrian University of Athens Medical School, Attikon Hospital, Athens, Greece

## Abstract

Oxidative stress (OS) is defined as a disturbance in the prooxidant-antioxidant balance of the cell, in favor of the former, which results in the antioxidant capacity of the cell to be overpowered. Excess reactive oxygen species (ROS) production is very harmful to cell constituents, especially proteins, lipids, and DNA, thus causing damage to the cell. Oxidative stress has been associated with a variety of pathologic conditions, including diabetes mellitus (DM), cancer, atherosclerosis, neurodegenerative diseases, rheumatoid arthritis, ischemia/reperfusion injury, obstructive sleep apnea, and accelerated aging. Regarding DM specifically, previous experimental and clinical studies have pointed to the fact that oxidative stress probably plays a major role in the pathogenesis and development of diabetic complications. It is postulated that hyperglycemia induces free radicals and impairs endogenous antioxidant defense systems through several different mechanisms. In particular, hyperglycemia promotes the creation of advanced glycation end-products (AGEs), the activation of protein kinase C (PKC), and the hyperactivity of hexosamine and sorbitol pathways, leading to the development of insulin resistance, impaired insulin secretion, and endothelial dysfunction, by inducing excessive ROS production and OS. Furthermore, glucose variability has been associated with OS as well, and recent evidence suggests that also hypoglycemia may be playing an important role in favoring diabetic vascular complications through OS, inflammation, prothrombotic events, and endothelial dysfunction. The association of these diabetic parameters (i.e., hyperglycemia, glucose variability, and hypoglycemia) with oxidative stress will be reviewed here.

## 1. Introduction

Oxidative stress (OS) is defined as a disturbance in the prooxidant-antioxidant balance of the cell, in favor of the former, so that the antioxidant capacity of the cell is overcome [1, 2], potentially leading to tissue injury [3]. It occurs due to an increased generation and/or reduced elimination of reactive species by the antioxidant defense system.

Reactive species are generally defined as chemical species containing unpaired electrons that subsequently increase the chemical reactivity of an atom or molecule [4]. In that way, they render the other molecules unstable and have the potential of damaging them, by initiating a chain of reduction-oxidation (redox) reactions [5].

Reactive species usually stem from the elements oxygen, nitrogen, sulfur, or halogen, which give rise to reactive oxygen species (ROS), reactive nitrogen species (RNS), reactive sulfur species, or reactive halogen species, respectively. The main obstacles in the proper and sound perception of the effects of ROS/RNS are the lack of a proper and universally accepted definition. These terms are vague, and since there are many ROS/RNS which are different in chemistry (some ROS/RNS are free radicals, but others are not; some free radicals are reactive, but others are not), or they can interconvert to one another [6], it is essential to explicitly specify the ROS used to properly interpret and discuss the role and effects of ROS in oxidative stress [2]. The most important reactive species formed in the human body are the oxygen derivatives. It is ironic that oxygen, an element indispensable for life, has detrimental effects on the human body under certain conditions [7]. Most of the potentially harmful effects of oxygen are due to the development and activity of these reactive oxygen species. They include the superoxide anion radical (O_2_−), the hydroxyl free radical (OH·), hydrogen dioxide (HO_2_·), hydrogen peroxide (H_2_O_2_), hypochlorous acid (HOCl), singlet oxygen (^1^O_2_), and various lipid peroxides. Also, transition metals such as iron and copper, nitric oxide (NO), and peroxynitrite (ONOO^−^) serve as free radicals [8, 9].

The human body continuously makes ROS during ordinary substantive metabolic processes. Mitochondria are the predominant source of ROS owing to the electron transport chain (ETC), but peroxisomes and the endoplasmic reticulum (ER) also contribute [10]. Actually, ROS may, under certain circumstances (low levels), have beneficial effects on the body, as they are used by the immune system as a way to assault and kill various pathogens [11]. In that way, they are considered to function in a beneficial way, modulating and maintaining key target functions by redox reactions, which is the essence of physiological oxidative stress, also called “oxidative eustress” [2, 12]. ROS are mainly produced during oxidative phosphorylation as a result of electron leak from the electron transport chain (ETC) located in the mitochondrial inner membrane [13] and are involved with the detoxification of xenobiotics by cytochrome P-450, with the elimination of microorganisms and cancer cells by macrophages and cytotoxic lymphocytes and with the manufacture of oxygenases (e.g., COX (cyclo-oxygenase) and LOX (lipo-oxygenase)) for the generation of prostaglandins and leukotrienes, which have many regulatory tasks. On the other hand, higher concentrations of ROS production lead to “adaptive stress” responses by the cell (via master switches, such as Nrf2/Keap1 (nuclear factor erythroid 2-related factor 2/Kelch-like ECH-associated protein 1) or NF-*κ*B (nuclear factor-kappa B)). Furthermore, in the event of an excessive load of oxidative stress production, called “oxidative distress,” it can lead to oxidative damage of the cells and provoke metabolic failure, compromising cell viability by inactivating enzymes of glycolysis, the Krebs cycle, and the ETC [12, 14, 15].

ROS can be composed by environmental and endogenous sources. Environmental sources include cigarette smoke, pollutants (such as ozone and nitrogen dioxide), ultraviolet light, ionizing radiation, and xenobiotics [7, 16]. Major endogenous sources include the NOX family of NADPH oxidases, complexes I and III of the mitochondrial ETC, the cytochrome P450-containing monooxygenase system, nitric oxide synthases (NOS), xanthine oxidoreductase, and myeloperoxidases [15]. In particular, NADPH oxidases are transmembrane proteins that transfer electrons from cytoplasmic NADPH across a biological membrane to molecular oxygen (O_2_) at the outer side of the membrane. NOX oxidases can function as electron transport chains, which transfer electrons first from NADPH(H+) to FAD leading to the formation of NADP+ and FADH2. Further, one electron is transferred to ferric ion (Fe^3+^) of the heme to produce ferrous ion (Fe^2+^). Afterwards, the electron is transferred across the cell membrane to molecular oxygen for its incomplete one electron reduction with the formation of superoxide anion radical. Other important endogenous sources of superoxide anion radical are enzymatic complexes of the mitochondrial ETC, in which more than 11 electron leakage sites have been found. The ETC is located in the inner mitochondrial membrane and composed of five enzymatic complexes (I-V). These complexes provide electron transportation from NADH(H+) or FADН2, which are formed in reactions of oxidation of various substrates in both mitochondrial matrix and the cytoplasm, to the final electron acceptor, molecular oxygen. The transportation of high-energy electrons along the respiratory chain is accompanied by release of their energy that is further transformed into the transmembrane electrochemical potential (Δ*μ*H) utilized for ATP biosynthesis in the process of oxidative phosphorylation. However, another process that may take place during the ETC functioning is incomplete one electron reduction of O_2_, which leads to formation of superoxide anion radical [17]. The produced superoxide anion radical can be released into either the mitochondrial matrix or the intermembrane space, depending on the site of its formation. Activities of the abovementioned ROS-producing enzymes can also be stimulated by arachidonic acid metabolites, such as prostaglandins, thromboxanes, and leukotrienes, produced by cyclooxygenases (COXs) and lipoxygenases (LOXs). In addition to NADPH oxidases and ETC complexes I and III, superoxide anion radical can be formed in the reactions catalyzed by xanthine oxidoreductase (XOR), in which hypoxanthine is irreversibly converted into xanthine, and further into uric acid in the final two reactions of purine catabolic pathway [18]. Superoxide anion radical can also be generated by enzymes containing transition metal ions as cofactors and heme as a coenzyme, i.e., metalloenzymes and hemoproteins, respectively [19]. ROS-producing hemoproteins include cytochrome P450- (CYP-) containing monooxygenase systems. Nitric oxide synthases (NOS) are heme-containing enzymes represented by three major isoforms (endothelial, neuronal, and inducible NOS) and generate NO (a primary type of RNS) in the cells, in the reaction of L-arginine conversion to L-citrulline. In addition to NO, all NOS isoforms can form superoxide anion radical as well [20]. Finally, myeloperoxidases (MPO), enzymes that catalyze the oxidation of substrates by hydrogen peroxide, can contribute to the formation of RNS (NO, nitrite ion, etc.), which contribute to their antibacterial activity [21].

ROS can assault various macromolecules in the body leading to damage of cells and homeostatic perturbations. Their targets include, among others, lipids, nucleic acids, and proteins [22]. Lipid peroxidation can lead to changes in membrane permeability and elasticity, as well as detrimental effects on membrane-bound proteins. Oxidation of nuclear or mitochondrial DNA can result in strand breaks, aberrant cross-linking, and DNA adducts (covalent bonding of DNA elements to chemical mutagens/carcinogens). Proteins (including crucial enzymes) may sustain oxidative damage at a variety of weak sites and be rendered biologically inert at high ROS concentrations [23], but, as stated above describing oxidative eustress, through the regulatory functions of low ROS concentrations, proteins may undergo oxidative modification of redox-sensitive residues, and this can cause changes in their activity and underlie alterations in their regulatory functions with no damage in structures and functions of the proteins and the cells [15].

Because of the likelihood of significant harm as a result of the ROS strike, various antioxidant defense systems have evolved to shield body tissues [3]. An antioxidant is a molecule competent of decelerating or preventing the oxidation of other molecules [24]. The body has several mechanisms to offset oxidative stress by producing antioxidants, either naturally created in the body (endogenous antioxidants) [25] or externally supplied through foods (exogenous antioxidants) [26]. Antioxidants preclude ROS-induced tissue injury by hindering the creation of radicals, scavenging them, or by promoting their decay. They cease the oxidative chain reactions by eliminating ROS intermediates and restraining other oxidation reactions by being oxidized themselves. The antioxidants include enzymes to decay peroxides, proteins to sequester transition metals, and a variety of compounds to “scavenge” ROS [27].

The endogenous antioxidant defense system consists of enzymatic and nonenzymatic antioxidants. Enzymatic antioxidants incorporate superoxide dismutase (SOD), catalase (CAT), glutathione peroxidase (GPx), and glutathione reductase (GSR) [28]. The nonenzymatic antioxidants are also partitioned into metabolic antioxidants and nutrient antioxidants. Metabolic antioxidants, belonging to endogenous antioxidants, are produced by metabolism in the body, such as lipoic acid, glutathione (GSH), L-arginine, coenzyme-Q10, melatonin, uric acid, bilirubin, metal-chelating proteins, and transferrin [29]. On the other hand, nutrient antioxidants, belonging to exogenous antioxidants, are compounds that cannot be created in the body and must be provided through foods or supplements, such as vitamin E, vitamin C, carotenoids, lycopene, trace metals (selenium, manganese, zinc), flavonoids, omega-3, and omega-6 fatty acids.

In particular, examining the most important food-borne antioxidants, vitamin E is the collective name for a set of eight related tocopherols and tocotrienols (alpha-, beta-, gamma-, and delta-tocopherol and alpha-, beta-, gamma-, and delta-tocotrienol), which are fat-soluble vitamins with antioxidant properties [30]. Of these, *α*-tocopherol has been most studied as it has the highest bioavailability, with the body preferentially absorbing and metabolizing this form. Its dietary sources are vegetable oils (corn, safflower, soybean, and sunflower), wheat germ oil, whole grains, nuts, cereals, fruits, eggs, poultry, meat, etc. It has been claimed that the *α*-tocopherol form is the most important lipid-soluble antioxidant and that it protects membranes from oxidation by reacting with lipid radicals produced in the lipid peroxidation chain reaction. This removes the free radical intermediates and prevents the propagation reaction from continuing. Vitamin C, also known as ascorbic acid, is a water-soluble vitamin that is essential for collagen, carnitine, and neurotransmitters' biosynthesis. It is believed to have antioxidant, antiatherogenic, anticarcinogenic, and immune-modulator effects. It works synergistically with vitamin E to quench ROS and also regenerates the reduced form of vitamin E. Natural sources of vitamin C are acid fruits (orange, lemon, grapefruit, pineapple, strawberry, etc.), green vegetables, tomatoes, etc. [31]. Carotenoids are a family of pigmented compounds that are present as microcomponents in fruits and vegetables and are responsible for their yellow, orange, and red colors. They are thought to be responsible for the beneficial properties of fruits and vegetables in preventing human diseases including cardiovascular diseases and cancer. They are important dietary sources of vitamin A and are thought to possess antioxidant activities as well [32]. Lycopene is a member of the carotenoid family of phytochemicals. It is a lipid-soluble antioxidant that is synthesized by many plants and microorganisms, but not by animals and humans and is responsible for the red color of many fruits and vegetables, such as the tomatoes. It is one of the most potent antioxidants and has been suggested to protect critical biomolecules including lipids, low-density lipoproteins (LDL), proteins, and DNA. Several studies have indicated that lycopene is an effective antioxidant and free radical scavenger [33]. Finally, flavonoids are polyphenolic compounds, present in most plants, with beneficial effects on human health, mainly due to their potent antioxidant activity [33]. Every plant contains a unique combination of flavonoids, which is why different herbs, all rich in these substances, have very different effects on the body. The main natural sources of flavonoids include green tea, grapes (red wine), apple, cocoa (chocolate), ginkgo biloba, soybean, curcuma, berries, onion, and broccoli.

In summary, oxidative stress results from the metabolic reactions that use chiefly oxygen in the body and characterizes a disruption in the balanced state of prooxidant/antioxidant reactions in living organisms, in favor of the prooxidant effects. ROS are well-acknowledged for playing a dual role as both injurious (oxidative distress) and advantageous (oxidative eustress) species. ROS and RNS are typically created by firmly controlled enzymes, and their overproduction results in a harmful process (oxidative distress) that can be a central mediator of injury to cell constituents, including lipids and membranes, proteins, and DNA. Because of this, oxidative stress is postulated to be involved in many human diseases, such as diabetes mellitus (DM), cancer, arthritis, inflammation, and coronary heart disease, as well as in the aging process. In contrast, positive effects of ROS/RNS arise at low/moderate concentrations and entail physiological adaptive roles in cellular responses to injurious stimuli (oxidative eustress), for example, in defense versus infectious agents, in the function of several cellular signaling pathways, and in the induction of a mitogenic response. The “two-faced” character of ROS is clearly substantiated [23].

The dysglycemia of diabetes mellitus can be portrayed as the glycemic triumvirate with its 3 main components: the persistent chronic (ambient) hyperglycemia, glucose variability, and hypoglycemic incidents [34]. The individual contributions of these glycemic disorders to the total risk of diabetic complications and the mode of their actions remain a topic of discussion. This review will examine the available evidence for the association between diabetes mellitus and oxidative stress, and especially the individual contribution of the three glycemic indices (hyperglycemia, glucose variability, and hypoglycemia) on the maintenance of “redox homeostasis” in people with diabetes and their involvement in the pathogenesis of diabetic complications.

## 2. Diabetes and Oxidative Stress

The prevalence of DM is rising and is achieving epidemic proportions. Recent data made available by the International Diabetes Federation (IDF) indicate that 463 million people were diagnosed with diabetes worldwide in 2019, and it is estimated that this number will increase to 700 million by 2045, the preponderance of who will be diagnosed with type 2 diabetes (T2D) [35]. Since DM is associated with augmented risk of micro- and macrovascular complications (such as retinopathy, neuropathy, and nephropathy, as well as cardiovascular disease (involving heart, cerebrovascular and peripheral arteries)), the projected increased incidence will result in higher medical care costs [36], reduced quality of life [37], and increased mortality [38].

Increased OS has been thought to be one of the key sources of the hyperglycemia-induced triggers of diabetic complications [39, 40]. On the other hand, OS has been alluded to the pathogenesis of diabetes per se [41], by leading to insulin resistance, dyslipidemia, *β*-cell dysfunction, and impaired glucose tolerance [42, 43]. As a matter of fact, even at the prediabetic state, visceral fat and superficial adipose tissue overexpress different cytokines (such as tumor necrosis factor-*α* (TNF-*α*) and interleukin 6 (IL-6)) and downregulate sirtuins (antiapoptotic proteins) that leads to increased inflammation and oxidative stress, which is associated to downregulation of mitochondrial biogenesis [44]. This may affect the cardiovascular functions in patients with diabetes vs. normo-glycemic individuals, leading to an altered myocardial performance and to the development of heart damage [45, 46]. Furthermore, it has been shown that prediabetes increases inflammatory burden in pericoronary adipose tissue as well, which also contributes to the increased CVD risk of these persons [47].

In diabetes, OS seems largely to be triggered by both a higher production of free radical species as well as a sharp decline in antioxidant defenses [48, 49]. The possible causes of OS might be the auto-oxidation of glucose [50, 51], swings in redox balances, decreased tissue concentrations of low molecular weight antioxidants (such as diminished GSH and vitamin E) [52], and impaired activities of antioxidant defense enzymes (such as SOD and CAT) [53]. This increase in ROS generation and the decrease in the activity of antioxidant defense systems due to hyperglycemia are thought to be largely responsible for the occurrence of diabetic complications [54, 55]. All three diabetic indices mentioned above (hyperglycemia, glucose variability, and hypoglycemia) [34] have been thought to play a role in the development of OS.

### 2.1. Hyperglycemia and Oxidative Stress

Hyperglycemia operates via several mechanisms to cause increased OS in DM and lead to vascular complications. Four core hypotheses have been proposed for the link between hyperglycemia-induced OS and complications [40]: “increased polyol (sorbitol) pathway flux, increased advanced glycation end-product (AGE) formation, activation of protein kinase C (PKC) isoforms, and increased hexosamine pathway flux”. They all seem to be involved in a vicious circle of a single hyperglycemia-induced process of ROS (superoxide) overproduction by the mitochondrial electron-transport chain and activation of these pathways. It has been also shown that endoplasmic reticulum (ER) stress plays an important role in oxidative stress, as it is also a source of ROS [56]. The tight interconnection between both organelles through mitochondrial-associated membranes (MAMs) means that the ROS generated in mitochondria promote ER stress as well. A brief description of these four mechanisms and their association with OS will follow.

#### 2.1.1. Increased Polyol (Sorbitol) Pathway Flux

**Figure 1:**
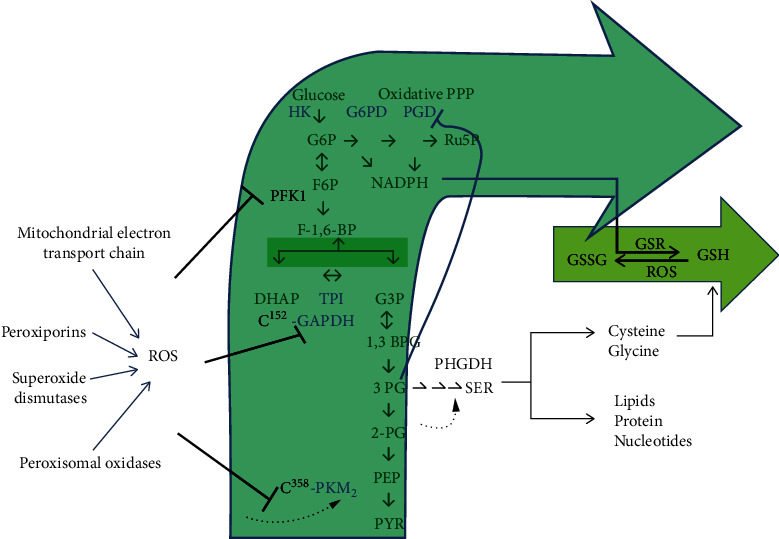
Reactive oxygen species- (ROS-) mediated inhibition of glycolysis reroutes flux into the oxidative arm of the pentose phosphate pathway. The enzymes inhibited by ROS are shown. ROS inhibits phosphofructokinase 1 (PFK1) and inactivates glyceraldehyde 3-phosphate dehydrogenase (GAPDH) and the pyruvate kinase isoform PKM2 by directly targeting cysteine residues. Thus, this glycolytic inhibition promotes flux into the oxidative pentose phosphate pathway (PPP) to produce NADPH and fuel cellular antioxidant systems (for example NADPH is consumed by glutathione reductase (GSR) to recycle oxidized glutathione (GSSG)). PKM2 inhibition is unique in that it allows for a diversion of flux into the serine synthesis pathway. Serine not only contributes to the synthesis of macromolecules but is also a precursor for glutathione (GSH). Serine synthesis is activated by a buildup of 2-phosphoglycerate (2PG), which prevents 3-phosphoglycerate- (3PG-) induced inhibition of the oxidative pentose phosphate arm. PPP: pentose phosphate pathway; HK: hexokinase; G6PD: glucose-6 phosphate dehydrogenase; PGD: phosphogluconate dehydrogenase; G6P: glucose-6-phosphate; Ru5P: ribulose-5-phosphate; F6P: fructose-6-phosphate; NADPH: nicotinamide adenine dinucleotide phosphate; PFK1: phosphofructokinase 1; F1,6 BP: fructose 1,6 bisphosphate; DHAP: dihydroxyacetone phosphate, TPI: tirose phosphate isomerase; G3P: glyceraldehyde-3-Phosphate; GAPDH: glyceraldehyde-3 phosphate dehydrogenase; 1,3 BPG: 1,3-bisphosphoglycerate; 3 PG: 3-phosphoglycerate; 2-PG: 2-phosphoglycerate; PEP: phosphoenolpyruvate; PYR: pyruvate; PKM2: pyruvate kinase isoform M2; PHGDH: 3-phosphoglycerate dehydrogenase; ROS: reactive oxygen species; GSSG: glutathione disulfide (oxidized glutathione); GSR: glutathione reductase; GSH: glutathione (reduced glutathione); SER: serine.

**Figure 2:**
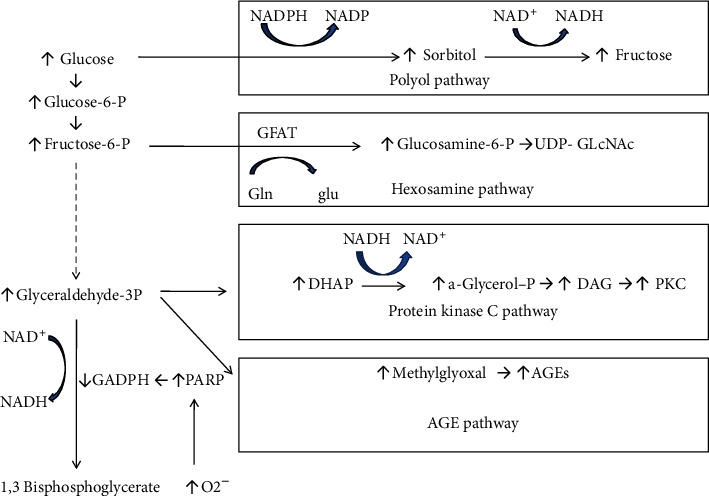
Mitochondrial overproduction of superoxide during hyperglycemic conditions activates four major pathways of hyperglycemic damage by inhibiting GAPDH. NADPH: nicotinamide adenine dinucleotide phosphate; NADH: nicotinamide adenine dinucleotide; GAPDH: glyceraldehyde-3 phosphate dehydrogenase; PARP: poly-ADP-ribose polymerase; GFAT: glutamine:fructose-6-phosphate amidotransferase; UDP-GlcNAc: UDP-N-acetylglucosamine; DAG: diacylglycerol; AGE: advanced glycation end-product.

The polyol pathway (or sorbitol-aldose reductase pathway) is a two-step process that converts glucose to fructose [57]. In this pathway, glucose is reduced to sorbitol, which is afterward oxidized to fructose. Aldose reductase (AR) is the first enzyme involved. It has a low affinity (high Km) for glucose, and at the normal glucose concentrations found in people without diabetes, metabolism of glucose by this pathway is negligible. But in a hyperglycemic setting (as occurs in uncontrolled diabetes), hexokinase (HK), the rate-limiting enzyme of the common glycolytic pathway (Figure 1), gets saturated and the surplus of glucose enters the polyol pathway, where AR reduces it to sorbitol (Figure 2). This reaction oxidizes NADPH (nicotinamide adenine dinucleotide phosphate) to NADP^+^. Sorbitol dehydrogenase (SDH) can then oxidize sorbitol to fructose, which produces NADH (nicotinamide adenine dinucleotide) from its oxidized form NAD^+^ [58]. Hexokinase can restore the molecule to the glycolysis pathway by phosphorylating fructose to form fructose-6-phosphate. However, in uncontrolled diabetes with high blood glucose—more than the glycolysis pathway can cope with—the reaction is altered towards the creation of sorbitol [59].

Many mechanisms have been proposed to explain the potentially harmful effects of hyperglycemia-induced increases in polyol pathway flux. These include sorbitol-induced osmotic stress, decreased Na^+^/K^+^-ATPase activity, increase in cytosolic NADH/NAD^+^, and decrease in cytosolic NADPH [40]. It seems that the latter is the most important [58]. NADPH, which is required for the preservation of the antioxidant reduced glutathione (GSH), is oxidized to NADP^+^ by the reduction of glucose to sorbitol in the AR pathway [60], and thus, the availability of intracellular NADPH is decreased. NADPH (a cofactor of NADPH-oxidases, the major ROS-generating system) is critically important, as it provides the reducing power that fuels the protein-based antioxidant systems and recycles oxidized glutathione. Furthermore, the antagonism between AR and glutathione reductase (GSR) for the NADPH cofactor further diminishes intracellular GSH [61]. GSH diminution regulates levels of cellular ROS production and accrual. Also, an increased ratio of NADH/NAD^+^ is connected with accelerated oxidation of sorbitol to fructose by NADH-dependent SDH [40]. The produced fructose can become phosphorylated to fructose-3-phosphate, which in turn can be broken down to 3-deoxyglucose and 3-deoxyglucosone. These two compounds are strong glycating agents that can glycate proteins and can result in the production of advanced glycation end-products (AGEs) [62], which, as mentioned below, are major pathogenic mediators of almost all diabetic complications. The NADH molecules are eventually transported to the mitochondria and oxidized by the respiratory chain reaction that results in the production of superoxide and other ROS, thus inciting oxidative damage to tissues [63, 64]. However, it has not been conclusively determined that activation of the polyol pathway in humans damages the vasculature [59].

#### 2.1.2. Increased Formation of Advanced Glycation End-Products

Advanced glycation end-products (AGEs) are proteins or lipids that become glycated as a result of exposure to sugars [65]. AGEs are formed through the Maillard reaction, which is the nonenzymatic reaction between the free amino groups of proteins and carbonyl groups of reducing sugars or other carbonyl compounds [66]. During this reaction, glucose (or other reducing sugars such as fructose, galactose, and xylulose) reacts with a free amino group of biological amines to develop an unstable compound, the Schiff base. This subsequently undergoes a rearrangement to a more stable product, the Amadori product [67], from which, in a later stage of glycation, irreversible compounds (the AGEs) are formed [68]. AGEs are produced not only from glucose but also from the dicarbonyl compounds produced from the autoxidation and the degradation products of glucose, such as glyoxal, methylglyoxal, and 3-deoxyglucosone, or *α*-hydroxy aldehydes such as glyceraldehyde and glycolaldehyde [69, 70]. In the case of chronic hyperglycemia, AGEs are actively produced and accumulate in the circulating blood and various tissues, contributing to vascular complications in diabetes [71].

Glycation of proteins results in interference with their standard functions by perturbing molecular conformation, modifying enzymatic activity, and interfering with receptor functioning [72]. AGEs form intra- and extracellular cross-linking with proteins and some other endogenous vital molecules, including lipids and nucleic acids, play a significant role in the occurrence of diabetic complications.

AGEs interrelate with plasma membrane-localized receptors (RAGEs) to modify intracellular signaling, gene expression, the liberation of proinflammatory molecules, and free radicals [73, 74]. RAGE has three splice variants of full-length RAGE, an N-terminal variant that does not contain an AGE-binding domain, a soluble receptor for advanced glycation end-product (sRAGE), and a C-terminal splice variant that does not include transmembrane and effector domains [75].

AGEs accelerate the expression of RAGEs, and they play an important role in the development of diabetic vascular complications through various mechanisms [58, 70]. By altering the function of modified intracellular proteins or of extracellular matrix components (which interact abnormally with other matrix components and with matrix receptors (integrins) that are expressed on the cells' surface) or by promoting the attachment of AGE-modified plasma proteins to AGE receptors on cells such as macrophages, vascular endothelial cells (ECs), and vascular smooth muscle cells, it stimulates the production of ROS. These, in turn, activate the pleiotropic transcription factor, nuclear factor-kappa B (NF-*κ*B), and increase expression of cell adhesion molecules, such as vascular cell adhesion molecule-1 (VCAM-1) in the vascular endothelial cells [76], causing numerous pathological changes in gene expression and giving rise to the pathogenesis of diabetic complications [77]. Furthermore, AGEs advance the production of the vascular endothelial growth factor (VEGF), leading to a rise in blood vessel permeability and the stimulation of neovascularization [78]. Also, circulating AGEs appear to react directly with lipoproteins, especially low-density lipoproteins (LDL), inducing structural alterations and damaging the mechanisms of LDL receptor-mediated particle removal at tissue level [79]. In patients with diabetes, RAGE expression is accelerated in atherosclerotic lesions in proportion to aggravation of blood sugar regulation [80, 81]. It has also been shown that the serum levels of the soluble form of RAGE (sRAGE) were significantly higher in type 2 DM patients compared to nondiabetic subjects and were positively associated with the presence of coronary artery disease [82]. AGEs are known to promote not only platelet aggregation but also the blood coagulation cascade through tissue factor production. The thrombotic tendency induced by AGEs is considered the cause of acute coronary syndromes, such as unstable angina or acute myocardial infarction through atheroma rupture and subsequent thrombogenesis in the coronary artery [68].

#### 2.1.3. Activation of Protein Kinase C (PKC) Isoforms

The PKC family is comprised of at least 11 isoforms, 9 of which are triggered by the lipid second messenger diacylglycerol (DAG) [83], which is enhanced by intracellular hyperglycemia [84]. Furthermore, oxidants, such as H_2_O_2_ [85], and mitochondrial superoxide induced by elevated glucose levels [86] can also trigger PKC in a distinct manner to DAG. Activation of PKC has several pathogenic results, by affecting the expression of endothelial nitric oxide synthase (e-NOS), endothelin-1 (ET-1), vascular endothelial growth factor (VEGF), transforming growth factor-*β* (TGF-*β*), and plasminogen activator inhibitor-1 (PAI-1), all of which play a role in vascular disorders. Also, it has been associated with activation of NF-*κ*B (which connects hyperglycemia-induced oxidative stress to inflammation [87]) and NAD(P)H oxidase [40]. In that way, PKC has been associated with vascular modifications such as increases in permeability, contractility, extracellular matrix synthesis, cell growth and apoptosis, angiogenesis, leukocyte adhesion, and cytokine activation and inhibition. These perturbations in vascular cell homeostasis caused by different PKC isoforms (PKC-*α*, -*β*1/2, and PKC-*δ*) are linked to the development of pathologies affecting large vessel (atherosclerosis, cardiomyopathy) and small vessel (retinopathy, nephropathy, and neuropathy) complications in DM [88, 89].

#### 2.1.4. Increased Hexosamine Pathway Flux

The hexosamine biosynthesis pathway (HBP) is a relatively insignificant limb of glycolysis, usually accounting for only 2–5% of the total glucose metabolism. Under hyperglycemic conditions, however, it can instigate posttranslational protein alterations by glycosylation and synthesis of glycolipids, proteoglycans, and glycosylphosphatidylinositol anchors [90]. In this pathway (Figure 2), fructose-6-phosphate is converted to glucosamine-6-phosphate, catalyzed by the first and rate-limiting enzyme, glutamine:fructose-6-phosphate amidotransferase (GFAT). The chief end-product is UDP-N-acetylglucosamine (UDP-GlcNAc) [91], which grants substrates for reactions such as proteoglycan synthesis and the formation of O-linked glycoproteins. Overalteration by this glucosamine frequently gives rise to pathologic transformations in gene expression [59, 92] and has been associated with some of the metabolic consequences of persistent hyperglycemia to promote the complications of diabetes [93, 94]. The reversible O-GlcNAc alteration of proteins by increased HBP activity could cause insulin resistance and the complications of diabetes [95]. Of particular relevance to diabetic vascular complications is the inhibition of endothelial nitric oxide synthase (e-NOS) activity in arterial endothelial cells by O-GlcNAcylation [96].

#### 2.1.5. A Common Mechanism of the Major Pathways' Activation

All these major pathways implicated in the pathogenesis of diabetic complications are activated by a single upstream incident, overproduction of reactive oxygen species (superoxide) [97], caused by the hyperglycemic intracellular setting (Figure 2) [40, 58, 59]. Specifically, in cells with high intracellular glucose concentration, there is more glucose-derived pyruvate being oxidized in the tricarboxylic acid (TCA) cycle, escalating the flux of electron donors (NADH and FADH2) into the electron transport chain. As a result, the voltage gradient across the mitochondrial membrane raises until a critical threshold is achieved. At this point, electron transfer inside the mitochondrial electron transport chain causes the leak of one electron to be transferred to molecular oxygen, thereby generating superoxide. Hyperglycemia also diminishes the activity of the key glycolytic enzyme glyceraldehyde-3 phosphate dehydrogenase (GAPDH) [98], and afterward, the level of all the glycolytic intermediates that are upstream of GAPDH increases (Figure 2). Augmented levels of the upstream glycolytic metabolite glyceraldehyde-3-phosphate activate the AGE formation pathway (because the chief intracellular AGE precursor methylglyoxal is formed from glyceraldehyde-3 phosphate) and also the classic PKC pathway (since the activator of PKC, diacylglycerol, is also formed from glyceraldehyde-3 phosphate). Further upstream, amounts of the glycolytic metabolite fructose-6 phosphate increase, which enhances flux through the hexosamine pathway. Finally, inhibition of GAPDH boosts up intracellular amounts of glucose, which enhances flux through the polyol pathway. The hindering mechanisms act through the poly-ADP-ribose polymerase (PARP) pathway, which alters GAPDH through polymers of ADP-ribose. Hyperglycemia provokes overproduction of ROS [99] and DNA single-strand cracks [100], both of which can stimulate PARP [101], thereby resulting in an alteration of GAPDH and a decrease of its activity [102] (Figure 2).

At the same time, metabolism has also evolved to respond to such ROS stresses in an adaptive manner. Frequently, the mechanism revolves around thiol-based switches that allow the cell to rewire metabolism in a way that promotes an antioxidant response independent of transcriptional or signaling pathways. Cells tune glycolytic metabolism to cope with oxidative damage by diverting glycolytic flux into NADPH-generating processes (Figure 1). The oxidative pentose phosphate pathway (ox-PPP), which produces ribose-5-phosphate, a precursor for nucleotide synthesis, is traditionally considered the predominant producer of cellular NADPH and is thus critical for antioxidant defense [103, 104]. Figure 1 depicts the sites whereupon ROS-mediated inhibition of glycolysis reroutes flux into the oxidative arm of the pentose phosphate pathway, to produce more NADPH and maintain cellular reducing power.

In summary, in today's environment, an overabundance of calories through food intake, combined with an inactive way of life in people with DM, results in high glucose (and fatty acid) accrual within the muscle, adipose tissue, and pancreatic cells [105], which provokes generation of ROS. ROS can act as signaling molecules, but when their production is exacerbated, they induce mitochondrial dysfunction and a decrease in ATP production. This stimulates PARP, lowers GAPDH activity, plays a significant role in increasing flux of the polyol pathway, stimulates PKC, raises intracellular production of AGEs, and overexcites the hexosamine pathway. This mechanism provides a refined link between hyperglycemia-induced oxidative stress and diabetic complications.

Given this pivotal role of hyperglycemia and oxidative stress on the development of diabetic complications, it is reasonable to assume that therapeutic strategies targeting these biological mechanisms could be pivotal to manage diabetes and prevent its serious complications. Thus, antidiabetic therapeutic agents, both pharmacologic and nonpharmacologic (e.g., diet and exercise), with combined hypoglycemic and antioxidant capacities, are thought to be better suited for preventing complications. In fact, all major classes of hypoglycemic agents have been investigated not only for their glucose-lowering but also for their antioxidant capacities as well [106].

In particular, metformin, the first-line antidiabetic medication in most persons with type 2 DM, which is an activator of AMP-activated protein kinase (AMPK) and suppresses hepatic glucose synthesis, improves insulin sensitivity by enhancing insulin-stimulated peripheral glucose uptake. Emerging evidence suggests that metformin boasts both direct and indirect antioxidant and anti-inflammatory properties, which may be contributing to its CVD protective effects [107]. Several mechanisms that explain metformin's beneficial actions have been proposed, including NF-*κ*B inhibition, NO production increment, and inhibition of AGEs formation. The antioxidative effect of metformin may be related to the reduction of diacylglycerol (DAG) levels, inhibition of PKC translocation to the cellular membrane, and suppression of the NADPH oxidase activity, leading to reduced ROS production [108]. In obese mice fed with a high-fat diet, treatment with metformin improved endothelial function by reducing endoplasmic reticulum stress and superoxide production and by increasing NO bioavailability [109]. Metformin has also been shown to directly inhibit ROS production from complex I (NADH: ubiquinone oxidoreductase) of the mitochondrial ETC [110] and to increase the AMP/ATP ratio. A recent study demonstrated that metformin treatment ameliorated high glucose-induced beta cell dysfunction by decreasing intracellular ROS production [111]. Even in prediabetic persons treated with metformin therapy, it was shown that their abdominal fat tissue presented higher sirtuin-6 (SIRT-6) expression and lower NF-*κ*B, PPAR-*γ*, and SREBP-1 expression levels, compared to a prediabetic control group [112]. Of note, it is known that SIRT6 activity is negatively regulated through reactive nitrogen species-mediated tyrosine nitration during oxidative stress [113], and at baseline, obese prediabetic patients show higher values of inflammatory and oxidative stress markers, and lower values of SIRT6 tissue protein expression than normoglycemic subjects, with a beneficial effect exerted by metformin therapy [112].

Thiazolidinediones (TZDs), including rosiglitazone and pioglitazone, are a class of drugs known to improve insulin sensitivity in peripheral tissues by binding to and activating the peroxisome proliferator-activated receptor gamma (PPARg). This receptor is involved in the regulation of expression of insulin-sensitive genes, which are crucial to glucose and lipid metabolism. Apart from the hypoglycemic effects, TZDs have shown an ability to modulate inflammatory, oxidative, and vascular functions. In *in vitro* and *in vivo* studies, pioglitazone protects against oxidative stress, reduces blood pressure, and decreases vascular cell adhesion molecule-1 (VCAM-1) expression on endothelial cells through modulation of NF-*κ*B activity via a PPARa-dependent mechanism [114]. It is postulated that these hypoglycemic and antioxidant effects, together with the anti-inflammatory effects of pioglitazone, have contributed to the beneficial cardiovascular effects seen in the PROACTIVE (Prospective Pioglitazone Clinical Trial in Macrovascular Events) trial [115].

Regarding insulin and sulphonylureas, in patients with type 2 DM, insulin treatment only partially improved oxidative stress parameters, as evidenced by the elevated levels of thiobarbituric acid reactive substances and reduced erythrocyte GSH [116]. Treatment with gliclazide for 12 weeks, on the other hand, ameliorated oxidative stress better than did glibenclamide [117]. Since gliclazide is one of the few hypoglycemic agents with an antioxidant effect [118], this may have contributed to the delayed progression of diabetic nephropathy in the ADVANCE trial [119], although it produced no significant effect on the development or progression of retinopathy or macrovascular complications in that study.

The incretin-based therapies, which include DPP-IV inhibitors [120] and GLP-1 receptor (GLP-1R) agonists [121], are a new class of antidiabetic medications, which aim to ameliorate the “incretin defect” present in people with diabetes [122]. Over the last years, extrapancreatic protective effects, behind glucose-insulin control, have been suggested in distinct vascular conditions [123–125]. In addition to regulating glucose and metabolic control, GLP-1 has a potential beneficial effect on multiple pathways involved in atherogenesis. Although the mechanisms of vascular effect are still unclear, it seems that the protective action of GLP-1 may be related to an improvement in endothelial dysfunction through its anti-inflammatory and antioxidant effects [126]. Even in patients with diabetes and nonobstructive CHD, it has been shown that after a non-ST-elevation myocardial infarction, people previously treated with incretins had beneficial effects on all-cause mortality, cardiac death, and readmission for acute coronary syndrome, compared to people not previously treated with incretins [127]. Liraglutide exerts marked antioxidant and anti-inflammatory effects on vascular ECs by increasing NO production, with inhibition of PKC-a, NADPH oxidase, NF-*κ*B, and JNK signaling, while also leading to the overexpression of superoxide dismutase (SOD) and catalase protective antioxidant enzymes [128]. Furthermore, it has been reported that liraglutide protects against atherogenesis by the reduction of Ox-LDL-induced mitochondrial ROS in human aortic vascular smooth muscle cells [129]. Regarding DPP-IV inhibitors, studies have demonstrated antioxidant and anti-inflammatory effects in animal models of diabetic nephropathy and diabetic retinopathy [130–132]. Clinical studies have failed though to show any benefit of this class of medications on CVD protection in people with DM at increased CVD risk [120].

Finally, the sodium-glucose cotransporter-2 (SGLT-2) inhibitors are the latest class of antidiabetic substances introduced in our therapeutic armamentarium for glycemic control. They work by inhibiting the absorption of glucose from the proximal tubule of the kidney, hence causing glucosuria, and have shown extremely favorable effects regarding cardiac and renal protection in persons with DM [133]. Recently, a few studies indicated that SGLT-2 inhibitors may exert their CVD and renal protection via anti-inflammatory and antioxidative effects [134]. Dapagliflozin was found to attenuate the formation of atherosclerotic lesions, increase the stability of lesions, reduce the production of IL-1b by macrophage infiltration, and decrease mitochondrial ROS generation in mice. These effects may be associated with an inhibitory effect on the NLRP3 inflammasome in diabetic atherosclerosis, which provides further evidence for its benefits in diabetic patients [135]. Moreover, also in diabetic rats, empagliflozin was shown to improve hyperglycemia, reduce urinary excretion levels of tubular injury markers, decrease expression levels of oxidative stress biomarkers (AGEs and RAGE), and reduce inflammatory and fibrotic markers in the kidney, including MCP-1, ICAM-1, PAI-1, and TGF-*β* [134]. These data suggest that a blockade of SGLT-2 by empagliflozin might protect proximal tubular cells from glucotoxicity in diabetic nephropathy, partly via suppression of the AGE-RAGE-mediated oxidative stress generation. These effects may have contributed to the beneficial effects seen with these agents in clinical trials of CVD and renal protection [136].

### 2.2. Glycemic Variability and Oxidative Stress

The assessment of glycemia by determining HbA1c reflects the average blood glucose levels over around the previous 2-3 months but does not afford any information about the actual fluctuations of glucose [137]. Because of this, individuals with similar HbA1c values may actually have had wide variations in their blood glucose levels over that period. It has been proposed that repeated or large glucose swings may contribute to diabetes-related complications, unrelated to HbA1c degree [138]. Postprandial escalations in blood glucose, together with hypoglycemic events, are blamed for higher cardiovascular events in DM [139], and postprandial plasma glucose has been associated in some studies more strongly to cardiovascular disease than fasting plasma glucose [140]. Glycemic variability (GV), i.e., oscillations in blood glucose levels over time, can represent the existence of excess glycemic excursions and, consequently, the risk of both of these events (hyperglycemic spikes and hypoglycemic episodes) [141, 142] and has been associated with the presence and severity of CVD in persons with diabetes [143].

Glycemic variability is currently defined in many different ways, either as short-term (within-day and between-day variability) [142] or long-term GV, usually based on sequential determinations of HbA1c, fasting blood glucose or other degrees of glycemia over a longer period (months or years) [144]. In the past, short-term glycemic variability was computed from self-monitoring of blood glucose with finger-sticks during a few days, but this method has been largely replaced over the past few years by continuous glucose monitoring (CGM), performed with the use of special devices [145] that measure the interstitial glucose levels continuously over several days [146]. These methods address many of the limitations inherent in HbA1c and self-monitoring of blood glucose [147]. The best and most accurate way to evaluate GV is still debated though [148], and the clinical relationship between GV and diabetes complications is actually difficult to ascertain because different studies are very heterogeneous regarding their design and the ways they use to assess GV.

Several studies (albeit not all [149–151]) have shown a positive relationship between glycemic variability and diabetes complications, both macrovascular and microvascular [152], as well as total and CVD mortality in both types of diabetes [144, 153, 154]. These data are in line with evidence that glycemic variability adversely affects plaque stability [155], is related to subclinical coronary atherosclerosis [156], and prolongs corrected QT interval duration [157], being also associated with the development of cardiac autonomic neuropathy [158–160].

Oxidative stress has been incriminated as the underlying mechanism for these effects of GV [161, 162] by the stimulation of superoxide production together with NADPH oxidase [163]. Interestingly, intermittently increased glucose levels have been shown to produce more oxidative stress than constantly elevated levels, since markers of inflammation, a well-recognized sign of oxidative stress, have been observed to increase in response to sporadically elevated glucose levels. In a study contrasting the consequences of inconsistent vs. constant glycemic conditions on cultured human kidney cells, it was noted that production of the inflammatory cytokines, transforming growth factor-*β* (TGF-*β*), and insulin-like growth factor-binding protein-3 (IGFBP-3) increased to a larger degree when exposed to erratic glucose concentrations compared with constant hyperglycemic states [164].

In other experimental studies, erratically high blood glucose rather than constant high blood glucose exposure was again shown to have harmful consequences [161, 165, 166]. In *in vivo* and *in vitro* studies, GV was associated with greater ROS production and vascular damage, compared to chronic hyperglycemia [162]. Intermittent high glucose levels (5 and 20 mmol/L (90 and 360 mg/dL) every 24 hours) induced ROS generation, which led to increased cellular apoptosis in human umbilical vein endothelial cells, compared with a constant high glucose setting (20 mmol/L (360 mg/dL)) [161, 165, 167]. *In vivo*, Horvath et al. investigated the effect of GV on oxidative stress and endothelial function in streptozotocin- (STZ-) induced diabetic rats [166]. Diabetic rats treated with intermediate long-acting insulin (insulin glargine) to accomplish stable normalization of blood glucose levels or long-acting insulin (ultralente insulin) once every other day, to generate “glycemic fluctuations” for 14 days, showed differing results of ROS production. Nitrotyrosine levels and endothelial dysfunction were elevated in diabetic rats with “glycemic swings” compared to rats with stable normalization of blood glucose.

Studies in humans have also substantiated these results. The effect of GV on oxidative stress and endothelial function in healthy controls and patients with T2D was examined with a euinsulinemic hyperglycemic clamp, to compare three different glycemic profiles over 24 hours: (1) 10 mmol/L (180 mg/dL) persistently, (2) 15 mmol/L (270 mg/dL) persistently, and (3) 5 and 15 mmol/L (90 and 270 mg/dL) every 6 hours (“glycemic swings”) [168]. It was found that GV produced greater endothelial dysfunction and oxidative stress, assessed by plasma 3-nitrotyrosine and 24-hour urinary excretion rate of 8-iso-PGF2a compared with persistent, either 10 or 15 mmol/L (180 or 270 mg/dL), glucose. These changes were overturned by concurrent infusion of vitamin C, suggesting that oxidative stress was the cause of endothelial dysfunction.

The association between GV and oxidative stress has also been investigated using CGM. A strong positive correlation between mean amplitude of glycemic excursions (MAGE, a marker of GV assessed by CGM) and a marker of oxidative stress (24-hour urinary excretion rate of 8-iso-PGF2a) was found in 21 patients with T2D [169]. A significant association between GV and the 24-hour urinary excretion rate of PGF2a in 26 T2D patients treated with diet and/or metformin has also been reported [170]. Furthermore, evidence exists that hyperglycemia after recovery from hypoglycemia leads to deterioration of endothelial function and rising oxidative stress and inflammation both in healthy control individuals and patients with type 1 diabetes (T1D), but not when recovery from hypoglycemia is followed by normoglycemia [171].

However, as mentioned earlier, some studies have shown conflicting results. Siegelaar et al. reported no significant correlation between GV and 24-hour urinary excretion of PGF2a in patients with T2D, well-controlled with oral antidiabetic medications [150]. In another study, Wentholt et al. explored the association between GV, assessed by CGM, and oxidative stress, assessed by 24-hour urinary excretion of 8-iso-PGF2a, in 25 patients with T1D [172]. Although higher levels of 8-iso-PGF2a were detected in patients with T1D compared with healthy subjects, no significant association was found between GV and oxidative stress in these patients. Differences in medications and patients' characteristics and also the dissimilar methods used to determine oxidative stress (ELISA vs. tandem mass spectrometry for 8-iso-PGF2a measurement) may explain the inconsistent results among these studies.

### 2.3. Hypoglycemia and Oxidative Stress

The maintenance of normoglycemia during treatment of diabetes while also trying to avoid hypoglycemia is a major challenge for patients and treating physicians. It has also been shown that excessive short-term glycemic variability, especially in the presence of target HbA1c levels, can contribute to the risk of hypoglycemia in both types of diabetes [173, 174].

There is evidence that hypoglycemia may unfavorably influence cardiovascular risk in patients with diabetes [175], and this is one possible explanation for the lack of CVD prevention in trials of intensive glycemic control. The ACCORD study [176], for example, showed that struggling to attain a very ambitiously low glycemic goal (HbA1c < 6% (42 mmol/mol)) with intensive therapy resulted in a greater incidence of hypoglycemia, although this increase was not proven to be causally related to the augmented risk of cardiac death observed in the study [177].

Hypoglycemia produces a surge of physiologic effects that may involve generation of oxidative stress and cardiac arrhythmias, contribute to sudden cardiac death, and bring about ischemic cerebral damage [178], portraying several likely mechanisms through which acute and chronic incidents of hypoglycemia may increase CVD risk [179, 180].

Hypoglycemia stimulates the sympathoadrenal system, causing a prolific discharge of catecholamines that exercise overwhelming hemodynamic and hemorheological effects [181]. The fact that hypoglycemia results in platelet hyperaggregability [182] and augmentation in several factors involved in the coagulation cascade has been known for over two decades. Activated partial thromboplastin time is abridged; fibrinogen and factor VIII increase and platelet numbers descend in association with hypoglycemia. These effects have the aptitude to compromise endothelial function, blood flow, and tissue perfusion, endangering intravascular coagulation, and thrombosis [183]. The proinflammatory effects of hypoglycemia in humans with or without diabetes were lately examined by *ex vivo* stimulations of peripheral blood mononuclear cells (PBMCs) and monocytes obtained during hyperinsulinemic-euglycemic-hypoglycemic clamps in eleven healthy controls, ten patients with T1D and normal awareness of hypoglycemia (NAH), and ten patients with T1D and impaired awareness of hypoglycemia (IAH). Hypoglycemia increased leukocyte counts in healthy controls and patients with NAH, but not in patients with IAH. Leukocytosis was robustly associated with the adrenaline response to hypoglycemia. The production of proinflammatory cytokines from stimulated polymorphonuclear cells and monocytes was larger after hypoglycemia compared to euglycemia, although it was less prominent in patients with IAH [184].

Furthermore, several studies have shown an association between hypoglycemia and ROS production. In experimental cell cultures, complete glucose deprivation stimulated the creation of mitochondrial ROS and AMP-kinase in cultured human umbilical vein endothelial cells (HUVECs) [185]. Moreover, insulin-induced recurrent hypoglycemia (two episodes/day for two weeks) in STZ-induced diabetic rats led to an increase in malondialdehyde (MDA) levels (product of the oxidation of n-6 unsaturated fatty acids) and a reduction in aconitase activity, which is an indicator of oxidative stress in brain mitochondria [186].

Insulin-induced hypoglycemia in nondiabetic male subjects was associated with increased proinflammatory cytokines (TNF-*α*, IL-1*β*, IL-6, and IL-8), indicators of lipid peroxidation and ROS production [187]. The increases in inflammation markers and oxidative stress in this study were very striking, maybe because the method of induction of hypoglycemia was by a bolus intravenous injection, which led to a swift descend of blood glucose concentrations, resulting in a quick release of catecholamines and the stimulation of the inflammatory response.

Two other studies have corroborated that hypoglycemia produces an upsurge in proinflammatory mediators and platelet activation. Wright et al. [183] and Gogitidze Joy et al. [188] both used a hypoglycemic clamp, during which blood glucose levels were retained at 2.5 and 2.9 mmol/L (45 and 52.2 mg/dL), respectively. The former maintained hypoglycemia for 60 min while the latter maintained it for 120 min. The extended duration of hypoglycemia in the study by Gogitidze Joy et al. resulted in a more remarkable rise in proinflammatory mediators, even though blood glucose levels were not as low as in the study by Wright et al. In another study, Ceriello et al. used 2-hour hyperglycemic and hypoglycemic clamps, with or without the concurrent infusion of GLP-1, and quantified markers of oxidative stress (plasma nitrotyrosine and plasma 8-iso-PGF2a) and markers of inflammation (soluble intercellular adhesion molecule-1 (sICAM-1) and IL-6) [189]. It was established that hypoglycemia significantly increased both markers of oxidative stress and inflammation. The same results were found after 2 hours of hyperglycemia. The concurrent infusion of GLP-1 or vitamin C significantly attenuated these effects. Vitamin C was actually more effective, implying a causal role of oxidative stress in favoring the manifestation of endothelial dysfunction and inflammation during hypoglycemia since an antioxidant agent was beneficial in attenuating the detrimental hypoglycemia-induced effects. When GLP-1 and vitamin C were infused concurrently, the harmful effect of hypoglycemia was almost entirely offset.

## 3. Conclusions

The overproduction of reactive oxygen species (ROS) is very harmful to the cell. Many studies are linking oxidative stress with a lot of pathological conditions, including diabetes as well as other human diseases. It has been reported that oxidative stress is implicated in the pathogenesis of diabetes per se, but also plays a major role in the occurrence and evolution of diabetic complications in both types of diabetes.

All three glycemic indices (hyperglycemia, glycemic variability, and hypoglycemia) have been associated with ROS production. Various mechanisms associated with hyperglycemia, such as the production of AGEs, the activation of PKC, the accumulation of sorbitol, and the hyperactivity of the hexosamine pathway, lead to reactive oxygen species overproduction and a decrease in the endogenous antioxidant defense systems. Several other studies have pointed that glycemic variability (GV) compared to chronic hyperglycemia is associated with greater ROS production, leading to vascular damage, most likely acting through the same mechanisms as hyperglycemia, but also with the possible additional effects of hypoglycemia. Recent evidence suggests that hypoglycemia is implicated in the production of oxidative stress, inflammation, hypercoagulability, and endothelial dysfunction, all of which favor diabetic vascular disorders. However, the exact mechanism by which oxidative stress is associated with hypoglycemia and the precise mechanism by which oxidative stress specifically contributes to the development of diabetic complications are partly unknown and need to be elucidated in future studies.
